# Serological Study on Cytomegalovirus and Toxoplasma Gondii in Thalassemia Major Patients of Yazd, Iran

**Published:** 2015-07-20

**Authors:** M Moghimi, M Doosti, HA Vahedian-Ardakani, A Talebi, M Akhavan-Ghalibaf, A Najafi, MM Aminorroaya, Sh Yazdani, M Shayestehpour, H Bahrami, F Khodayari

**Affiliations:** 1 **Assistant Professor of Clinical and Surgical Pathology,Department of Pathology,Shahid Sadoughi،Yazd ،Iran**; 2 **Infectious and Tropical Diseases Research Center, Shahid Sadoughi University of Medical Sciences, Yazd, Iran.**; 3 **Department of Internal Medicine, Shahid Sadoughi University of Medical Sciences, Yazd, Iran.**; 4 **Infectious and Tropical Diseases Research Center, Shahid Sadoughi University of Medical Sciences, Yazd, Iran.**; 5 **Shahid Sadoughi hospital, Shahid Sadoughi University of Medical Sciences, Yazd, Iran.**; 6 **Department of Clinical Immunology, Shahid Sadoughi University of Medical Sciences, Yazd, Iran. Shahid Sadoughi hospital, Yazd, Iran**; 7 **Department of Virology, School of Public Health, Tehran University of Medical Sciences, Tehran, Iran.**

**Keywords:** Beta-Thalassemia, Cytomegalovirus, ELISA, Toxoplasma

## Abstract

**Background:**

Beta-thalassemia patients receive blood products from blood transfusion centers repeatedly. Blood transfusion can transmit Cytomegalovirus (CMV) and Toxoplasma gondii. The aim of this study was serological evaluation of these two infectious agents in thalassemia patients.

**Materials and Methods:**

In a cross-sectional study, the enzyme-linked immunosorbent assay (ELISA) testing was performed to detect IgM and IgG antibodies against CMV and Toxoplasma gondii in 96 thalassemia patients (under 18 years) and 144 healthy people. Data were analyzed by SPSS software and Chi-square test.

**Results:**

A significant difference was observed in CMVIgM antibody levels between test groups in women (p<0.05). The prevalence of CMV IgM, CMV IgG, Toxo-IgG, and Toxo IgM antibodies in thalassemia patients were 5.2%, 95.9%, 16%, and 0%, respectively.

**Conclusion:**

In all thalassemia patients, Cytomegalovirus IgG is higher than healthy people. In addition, CMV IgM antibodies are higher in female patients. Antibody screening (IgM) on blood products for detecting Cytomegalovirus is necessary, but for Toxoplasma gondii is not necessary in the Yazd transfusion center.

## Introduction

Thalassemia major is a severe anemia disease. This disorder occurs in children with two mutated genes that are inherited from each parent. These patients cannot produce normal hemoglobin and experience chronic fatigue (1, 2). Two major consequences of thalassemia are severe anemia and expansion of bone marrow in the body. These consequences lead to poor growth, impaired physical activities as well as other bone deformities, fragile bones and enlargement of the liver and spleen (3). The only treatments for severe anemia are regular blood transfusions and iron chelation therapy (2). Many contagious agents can be transmitted potentially through blood transfusions, including bacteria, viruses, and parasites (4). Considering frequent need of thalassemia major patients to blood

components, the chance of transmission of noted infectious agents in these patients is high (3).

Cytomegalovirus (CMV) is a DNA virus and a member of herpesviridae family (5). This virus is important in blood transfusion. In patients with a compromised immune system, CMV infection can cause lethal consequences (6). Universally, this virus is distributed with approximately 40-100% of the world's population (7). The prevalence of CMV antibodies in thalassemia major patient is higher than normal persons (8-10).

Toxoplasma is a parasite that transmits by contact with cats and their feces, eating under cooked meat, drinking raw milk or organ transplantation (11). Thirty to fifty percent of the world population is infected with toxoplasmosis (12). Overly, more than thirty percent of people in Iran are exposed to this parasite (13). This agent can transmit by blood transfusion and can danger in pregnant women and immune compromised people (14, 15). In people with weak immune system, especially as a result of HIV/AIDS, toxoplasmosis can lead to seizures and life-threatening illnesses such as encephalitis (16). 

Toxoplasma in healthy people sometimes is caused eye infections and an untreated case is caused blindness (17). Donors in Iranian blood transfusion centers are not screened for CMV and Toxoplasma infections. The aim of this study was the serological evaluation of Cytomegalovirus and Toxoplasma in thalassemia major patients.

## Materials and Methods

For performing this cross-sectional study, we were selected 144 healthy blood donors without any history of hematologic disease and repetitive transfusion (64 male and 80 female who were under 18 years old) and 96 beta-thalassemia major patients (45 male and 51 female who were under 18 years old) who received blood regularly from Yazd transfusion center. Blood samples were collected in volume of five ml from each participant. Then serums were analyzed for detecting CMV-IgM, CMV- IgG, Toxoplasma_IgM, and Toxoplasma_IgG antibodies. For this purpose, Enzyme Immunoassay test (ELISA) performed using Pishtaz Teb Diagnostics kits (Iran). One hundred µl of each control as well as diluted test sera were added into appropriate wells of the antibody coated plate. Two consecutive wells in the first strip as the blank and positive were considered. Plates were sealed with cardboard seal tightly and incubated for 30 minutes at 37˚C. Consequential steps carried out according to protocols of Pishtaz Teb Corporations. Finally, 100µl of stop solution added to the wells and absorbance read at 450 nm by ELISA reader. The specificity and sensitivity of Pishtaz Teb Diagnostics kit for ELISA test was 99% and 100%, respectively. Data were analyzed by SPSS software version 19 and Chi-square test was performed for evaluation of relationships.

## Results

The result of ELISA tests (IgM and IgG) for CMV and Toxoplasma is summarized in Table 1 and Table 2. Ninety six beta-thalassemia major patients, including 46.8% male and 48.2% female, were tested by ELISA method. CMV IgM and IgG antibodies were detected in 10% and 94.1% of patients, respectively. This amount for men was 0% and 97.7%, respectively. Toxoplasma IgM antibody was not detected in the studied population. Furthermore, 15.6% of women and 15.5% of men were seropositive for Toxoplasma IgG antibody. Data for the thalassemia patients according to the interval time between transfusions is showed in Table 3. Rate of seropositive patients was high, when the interval time between blood transfusions was 20 days. Sixty percent of thalassemia patients, received blood with 20 days interval between transfusions was seropositive for CMV IgM antibody. Data showed the statistical significant difference for IgG anti-Toxoplasma gondii and CMV IgM antibodies between the case and control groups (p<0.05).

**TableI:** The result of CMV Enzyme Immunoassay test (ELISA) for thalassemia patients and control groups

Test	Result	Male	Female	Total
CMV IgM seropositive	Thalassemia patients	0	5	5 (5.2%)
	Normal persons (control)	2	1	3 (2%)
	P value	> 0.05	0.036	> 0.05
CMV IgG seropositive	Thalassemia patients	44	48	92 (95.9%)
	Normal persons (control)	63	79	142 (98.6%)
	P value	> 0.05	> 0.05	> 0.05

**TableII:** The result of Toxoplasma Enzyme Immunoassay test (ELISA) for thalassemia patients and control groups

Test	Result	Male	Female	Total
Toxo IgM seropositive	Thalassemia patients	0	0	0
	Normal persons (control)	0	0	0
	P value	-	-	-
Toxo IgG seropositive	Thalassemia patients	7	8	15 (16%)
	Normal persons (control)	2	2	4 (3%)
	P value	< 0.05	< 0.05	0.004

**TableIII:** Data analysis of seropositive patients according to interval time between blood transfusions

test	interval time between transfusions (day)			
	15	20	25	30
CMV IgM seropositive	1 (20%)	3 (60%)	0	1 (20%)
CMV IgG seropositive	3 (3.3%)	65 (71.4%)	4 (4.3%)	19 (21%)
Toxo IgM seropositive	0	0	0	0
Toxo IgG seropositive	2 (13.3%)	10 (66.6%)	2 (13.3%)	1 (6.8%)

## Discussion

Because of the prevalence and circulating of Cytomegalovirus in Iranian population, the result of this study, like all of the previous studies, showed that rates of CMV IgG antibody in two groups of the study population, thalassemia major patients and normal peoples, were more than ninety percent (1-4). The receiving of blood for thalassemia major patient is frequently necessary. Therefore, this patients are exposed to risk of CMV infection transmission (5). The results of the present study reported that CMV IgM antibodies in thalassemia patients were higher than others. In 2007, Dr choobineh et al. was reported that CMV IgM antibodies in the Iranian thalassemia patients below 15 years were 12.9% (6, 7). No significant difference was found between male and female CMV IgM. However, all seropositive patients were women in our study. In another study, Iranian researcher showed that CMV IgM seropositivity for thalassemia patients of Tehran is 9.1% (3). IgM antibodies were positive in 0.04% of the normal group. It is essential to note that population rush had an important role in transmission of the virus. Tehran is more crowded than Yazd. Therefore, the virus circulates easily and transmits to the thalassemia patients by blood transfusion. Although, it seems that screening of blood donors for CMV IgM antibody is necessary and thalassemia patient must receive safer blood components.

Toxoplasma gondii is a microscopic parasite that infects cats and can be found in the soil (8). The infection can be acquired by ingesting unsanitary food or water, by handling contaminated cat litter, or by transmission from mother-to-child (9). Toxoplasmosis is detected in all countries, and seropositivity rates range from 6 percent in South Korea to 92 percent in Ghana (10). The prevalence of Toxoplasma infection varies with social class and hygiene levels. In France, the cultural preference of undercooked meat is caused high prevalence of toxoplasmosis. In the rural area of France, 47% of people were seropositive (11). Toxoplasma transmission can also be attributed to the type of lifestyle that individuals live in. A study was performed (in 2007) in Qalyubia Governorate of Egypt to measure the prevalence of toxoplasmosis in pregnant women. Fifty seven percent of disease cases were detected in the rural areas when the rate of infection for the urban areas was 46.5%. The rural areas of Egypt generally have higher levels of stray cats (12). Twelve percent of people in China, 6.7% in Korea and 23.9% in Nigeria are seropositive for Toxoplasma (13-15). A serological survey of anti-Toxoplasma gondii antibodies in beta-thalassemia major patients at Aydin's province of turkey showed that Toxo IgM antibody in patients is higher than normal peoples (5.5% versus 0%) (16).

In the previous research in Iran, Seropositivity for Toxoplasma had been reported as 48-74.6 in the north, 22-37% in the south,33-44% in northwest and 27-54% in central cities of Iran (17, 18). These studies mainly have been done on pregnant women. In 2014, Dr Jafari et al. evaluated the Toxoplasma antibodies among 375 blood donors in Zahedan. Twenty five percent of people were Toxoplasma IgG positive, but not reported Toxoplasma IgM positive (19). A serological study was performed in hemodialysis patients of Abadan and Khoramshahr cities of Iran in 2011 for toxoplasmosis. IgM antibodies were not detected in the control group, but 8.67% of hemodialysis patients were positive (20). In our study, Toxoplasma IgM antibody was not detected in any person of control or patient groups. IgG antibodies for Toxoplasma were detected in thalassemia patients higher than normal patients (16% versus 3%). The number of population of Yazd is low compared to other cities of Iran. Furthermore, the people of this city have high income and hygienic levels. Thus, the low prevalence this parasite in this area is logical. Thalassemia patients receive blood frequently. Therefore, these patients have IgG antibodies higher than others. Results showed that most thalassemia patients that received blood with twenty-day interval between transfusions were positive for CMV IgM antibody nevertheless, no significant difference was found in our study (p >0.05) regarding the relationship between transfusion intervals and the prevalence antibodies in patients.

## Conclusion

In countries and areas with low rate of toxoplasmosis, the transmission risk through blood transfusion is very low, and serologic testing for screening blood donors appears to be unnecessary. In all thalassemia patients, CMV IgG was higher than the control group. Additionally, CMV IgM antibodies were high in female patients. Antibody screening (IgM) on blood products for Cytomegalovirus is necessary, but for Toxoplasma gondii is not necessary in the Yazd transfusion center of Iran.

## Conflict of interest

The authors have no conflicts of interest.
